# Editorial: Microbial production of medicinally important agents

**DOI:** 10.3389/fmicb.2023.1274087

**Published:** 2023-09-20

**Authors:** Jia Zeng, Jixun Zhan, Xue Qiao, Ozkan Fidan

**Affiliations:** ^1^Thermo Fisher Scientific, San Jose, CA, United States; ^2^Department of Biological Engineering, Utah State University, Logan, UT, United States; ^3^Department of Pharmaceutical Analysis, School of Pharmaceutical Sciences, Health Science Centre, Peking University, Beijing, China; ^4^Department of Bioengineering, Abdullah Gül University, Kayseri, Türkiye

**Keywords:** microbial, fermentation, synthetic biology, medicinal agents, bioprocessing

Harnessing microbial systems as bio-factories for the production of medically significant agents presents a thriving avenue in pharmaceutical research. From manufacturing natural products, including potent secondary metabolites, to the sophisticated engineering of recombinant proteins, microbial production's contributions are manifold (Katz and Baltz, 2016). A salient trend is the rapid evolution of synthetic and molecular biology tools, which substantially enhance our capacity to manipulate microbial metabolism (Keasling, 2012; Ko et al., 2020). Furthermore, refinements in bioprocessing strategies have significantly improved the overall yield of microbial products, emphasizing the cost-effectiveness and efficiency of microbial production (Garcia-Ochoa and Gomez, 2009; Sharma et al., 2020). These advancements, in tandem with predictive technologies such as machine learning for optimal microbial strain selection and fermentation condition prediction, showcase this field's innovative trajectory.

Despite substantial progress, challenges remain, including post-modification, expression systems, and product complexity. Nevertheless, the ever-evolving realm of microbial engineering and biotechnology continues to offer promising solutions. As we embark on exploring medicinally significant agents derived from microbial sources in this Research Topic, we applaud the progress made thus far and eagerly anticipate future innovations in microbial production (Cui et al., 2020; McAdam et al., 2020; Srinivasan and Smolke, 2020; Tabatabaei et al., 2020; Guzior and Quinn, 2021; Kumar et al., 2022; Serra et al., 2023; Tariq et al., 2023). This Research Topic aims to provide crucial insights on two fronts: broadening the spectrum of essential biopharmaceutical applications and exploring effective solutions for improved production performance. Each of these areas underscores the potential that microbial production holds. Through this Research Topic, we aim to illustrate the advancements in enhancing microbial performance and inspire further innovations in microbial engineering.

The industrial manufacture of penicillin (Scriabine, 1999), a life-saving antibiotic first produced on a large scale during World War II, serves as a compelling example of microbial production. This biotechnological milestone paved the way for developing more advanced antibiotics. Similarly, research on amphiphilic aminoglycosides has demonstrated how chemical modifications can enhance antibiotics' effectiveness and broaden their applications, introducing our theme of performance enhancement (Takemoto et al.).

Broadening the spectrum of essential biopharmaceutical applications necessitates the exploration of new sources. We turn our gaze to the Arctic, where research on marine actinobacteria has unearthed new bioactive metabolites (Schneider et al.). This study demonstrates how untapped ecosystems can lead to the discovery of novel medicinal compounds, thereby expanding our theme of performance enhancement. Additionally, another review paper that focused on endophytes and marine microorganisms has unveiled potential glycosidase inhibitors, exemplifying the extensive range of medicinal compounds derivable from microbial sources (Wang et al.).

Optimization of the process occurs at various levels and stages. On the enzyme and protein level, the study of marine microbial carboxylesterase, E93, stands out (Li et al.). This enzyme can hydrolyze substrates challenging for human carboxylesterase, highlighting the advantages of microbial enzymes in medicinal applications and highlighting performance enhancement. Moreover, an investigation on Resuscitation-promoting factor B (RpfB) from *Rhodococcus* sp. has demonstrated the potential to boost microbial biological activity, with an increase of 18% in cell resuscitation, through condition optimization (Gong et al.).

Delving into the molecular level, two studies illustrate the potential of gene manipulation in augmenting microbial productivity. The reengineering of 7-dehydrocholesterol biosynthesis in *Saccharomyces cerevisiae* has achieved the highest titer reported to date (Wei et al.). Similarly, manipulating nutrient sources in Cordyceps mushroom cultivation influenced gene expression and transcriptional levels, resulting in a 34-fold increase in cordycepin production (Turk et al.).

The strategies employed in microbial production to enhance performance and productivity vary widely. Each example shared in this Research Topic outlines the myriad ways that microbial production can be optimized to produce medically significant agents. It becomes clear that the microbial world's microscopic components hold immense potential. The examples serve as both an inspiration and a guiding star, importance the success achieved and the path forward for future endeavors in the microbial production of medicinally important agents. With continual research and advancements, we can further unravel this potential to meet and overcome the medical challenges that lie ahead.

## Author contributions

JZe: Writing—original draft. JZh: Writing—review and editing. XQ: Writing—review and editing. OF: Writing—review and editing.
